# Knowledge, Attitudes, and Practices About Parkinson’s Disease Among the General Population of the Eastern Province of Saudi Arabia

**DOI:** 10.3390/healthcare13070795

**Published:** 2025-04-02

**Authors:** Meshari Lafi Alshammari, Aseel Hameed Al-Banaqi, Ahmad Sulaiman Almutairi, Sami Sharkan Alqahtani, Meshal Mohammed Alharbi, Esraa M. Haji, Mohammed Nazrul Islam, Mohammed Salem Alshammari, Ashfaq Ahmad

**Affiliations:** 1 Department of Pharmacy Practice, College of Pharmacy, University of Hafr Al Batin, Hafr Al Batin 39524, Saudi Arabia; ph.meshari70@gmail.com (M.L.A.); aseelhameed2229@gmail.com (A.H.A.-B.); a7md.5541@gmail.com (A.S.A.); samiqa1200@gmail.com (S.S.A.); m4al70x@gmail.com (M.M.A.); mnazrul@uhb.edu.sa (M.N.I.); moalshammari@uhb.edu.sa (M.S.A.); 2 Department of Pharmaceutical Chemistry, College of Pharmacy, University of Hafr Al Batin, Hafr Al Batin 39524, Saudi Arabia; emhaji@uhb.edu.sa

**Keywords:** Parkinson’s disease, knowledge, attitudes, practices, sociodemographic factors, gender differences

## Abstract

**Background**: Understanding how sociodemographic factors influence knowledge, attitudes, and practices (KAP) related to Parkinson’s disease (PD) is essential for developing targeted interventions. This study examines the associations between KAP components and variables such as gender, age, education, and marital status. **Methods:** A cross-sectional survey was conducted among the general population from October 2024 to February 2025. Data on sociodemographic characteristics and KAP related to PD were collected using structured questionnaires. The analysis was done by SPSS version 28. **Results:** Male participants demonstrated higher mean knowledge scores (4.16 ± 0.7) compared to females (3.93 ± 0.7), with a significant difference (*p* = 0.01). Participants aged 50–59 years had the highest mean knowledge scores (4.53 ± 0.8), while those aged 21–29 years had the lowest (3.85 ± 0.6), with a significant difference (*p* = 0.01). High school-educated participants exhibited the highest mean knowledge scores (4.51 ± 0.7), whereas those without formal education had the lowest (3.89 ± 0.5), with a significant difference (*p* = 0.01). Regarding attitudes, participants aged 40–49 years scored highest (8.24 ± 1.5), and those over 60 years scored lowest (7.10 ± 1.1), with a significant difference (*p* = 0.03). Single participants had higher attitude scores (8.16 ± 1.3) than married ones (7.60 ± 1.6), with a significant difference (*p* = 0.01). In practice, males scored higher (4.16 ± 0.7) than females (3.93 ± 0.7), with a significant difference (*p* = 0.01). Participants aged 50–59 years had the highest practice scores (4.53 ± 0.8), and those aged 21–29 years had the lowest (3.85 ± 0.6), with a significant difference (*p* = 0.01). **Conclusions:** Sociodemographic factors significantly influence KAP related to PD. Tailored educational interventions, considering these variables, are crucial for enhancing PD awareness and management. Our study indicates that the general population has moderate knowledge regarding PD. Most participants have a positive attitude regarding helping and supporting people who are suffering from PD. Educational attainment emerged as a critical determinant, with those having a high school education showing the highest mean scores in both knowledge and practices, underscoring the role of education in health literacy and proactive health behaviors. Furthermore, marital status influenced attitudes toward PD, with single participants displaying more positive attitudes than their married counterparts.

## 1. Introduction

Parkinson’s disease (PD) is a progressive neurodegenerative disorder characterized by motor symptoms such as tremors, rigidity, bradykinesia, and postural instability, as well as non-motor symptoms including cognitive impairment, mood disorders, and autonomic dysfunction [1]. As the second most common neurodegenerative disease after Alzheimer’s, PD poses significant challenges to healthcare systems worldwide, particularly in aging populations [2].

Annual incidence estimates for Parkinson’s disease (PD) fluctuate, with estimated rates between five and over thirty-five new cases per 100,000 individuals. The incidence climbs five to tenfold during the sixth and ninth decades of life. The probability of Parkinson’s disease escalates with advancing age. A meta-analysis of four North American cohorts revealed that prevalence increased from below 1% among individuals aged 45–54 to 4% in men and 2% in women aged 85 and older. Mortality rates in individuals with Parkinson’s disease are not considerably elevate during the initial ten years post-diagnosis compared to unaffected individuals, but they subsequently climb. The incidence of Parkinson’s disease is anticipated to more than double in the next two decades due to the ageing global population. The societal and financial burden of Parkinson’s disease will escalate if novel cures, treatments, or preventive strategies are not developed [3,4].

In Saudi Arabia, the prevalence of PD is expected to rise due to increasing life expectancy and demographic shifts, underscoring the need for greater public awareness and understanding of the disease [5]. In Saudi Arabia, the prevalence of PD is estimated at 27 per 100,000 individuals. Despite advancements in diagnosis and management, public awareness and understanding of PD remain limited [6].

The Eastern Province of Saudi Arabia, encompassing key cities such as Dammam, Al-Khobar, and Dhahran, has a unique demographic and cultural composition [7]. Comprehending the knowledge, attitudes, and practices (KAP) of PD in this region is crucial for formulating targeted educational initiatives and healthcare policies. Nevertheless, there is a scarcity of data specifically regarding the KAP related to PD within the general population in the Eastern Province.

This study seeks to assess the knowledge, attitudes, and practices of Parkinson’s disease within the general community of the Eastern Province of Saudi Arabia. This research aims to elucidate the current state of awareness regarding Parkinson’s disease (PD) by examining knowledge of its symptoms, risk factors, and treatment choices, as well as attitudes towards affected individuals and healthcare-seeking behaviors. The results will aid in the formulation of targeted interventions to boost public comprehension, mitigate misconceptions, and bolster support for those impacted by PD, hence enhancing their quality of life and overall health outcomes.

## 2. Materials and Methods

### 2.1. Study Design and Setting

This observational cross-sectional study was conducted from October 2024 to February 2025, in the Eastern Province of Saudi Arabia, using a validated questionnaire. The Eastern Province is one of the largest regions in Saudi Arabia, encompassing major cities such as Dammam, Al-Khobar, and Hafr Al Batin, which have well-established healthcare facilities, including specialized neurology and movement disorder centers. Additionally, the region has a diverse population and a growing aging demographic, which may contribute to an increasing burden of Parkinson’s disease.

### 2.2. Study Instrument and Translation

The survey instrument was developed based on the previous literature about the knowledge, awareness, attitude, and practices of PD [8,9]. Three researchers assessed the questionnaire tool to determine the suitability, relevance, clarity, and adequacy of the questions. The survey instrument had 25 questions, encompassing socio-demographic variables and knowledge regarding PD (nine items). A large score means better knowledge, practice, and attitude. The survey’s initial version was composed in English. A qualified translator subsequently rendered it into Arabic and subjected it to a back-to-forward translation process. Translators with sector-specific expertise and experience in interpreting surveys, whose native language was Arabic, conducted the forward translation. A second translator, a native English speaker, was engaged to translate the questionnaire back into the original language following the authors’ receipt of the initial translation.

The questionnaire was disseminated online to a random sample of individuals residing in various parts of the Eastern Province of Saudi Arabia. The questionnaire was disseminated via online platforms like WhatsApp, Telegram, Facebook, and emails. All participants were required to respond to all questions, and incomplete questionnaires were excluded from the study.

### 2.3. Ethical Considerations

This study received ethical approval from the local research ethics committee at Hafr Al-Batin University, with reference number HPO-05-FT-25/03. Within the framework of ethical considerations, confidentiality and informed consent were key priorities. Participants were assured of the strict confidentiality of their information throughout the study and were given the right to cease or withdraw their consent.

### 2.4. Data Collection Tools and Techniques

This study utilized a random sampling technique. Individuals who fulfilled the inclusion criteria were solicited to partake in the study. The study variables encompassed sociodemographic parameters, including age, gender, educational attainment, and nationality, alongside knowledge-related aspects such as awareness of Parkinson’s disease (PD), its signs and symptoms, risk factors, and related practices.

The structured interview questionnaire utilized in this study was designed based on an analysis of previous research and expert insights, as outlined in the appendix questionnaire [10]. The questionnaire underwent pre-testing with 30 individuals from the target group who were excluded from the study to verify its clarity, relevance, and validity (piloting study). Cronbach’s alpha yielded a result of 0.89, indicating a satisfactory level of reliability.

### 2.5. Data Analysis Plan

The data were analyzed using SPSS version 28 (IBM Corp., Armonk, NY, USA). Descriptive statistics such as frequencies and percentages were used to summarize the sociodemographic and knowledge-related variables. Inferential statistics such as *t*-tests and ANOVA were used to examine the associations between categorical variables. The variable’s significance cutoff was at the 0.05 level.

## 3. Results

### 3.1. Sociodemographic Characteristics of the Participants

The study surveyed 640 individuals from the Eastern Province of Saudi Arabia, with a majority being male (67.2%). Nearly half (46.3%) were aged 21–29, and 60.8% held postgraduate degrees. Students comprised 43% of the sample, while 39.7% were unemployed. Most participants (56.1%) had an income above 5000 SAR. Geographically, 72.2% resided in Hafr al-Batin. Marital status distribution showed 66.1% were married, 32% single, and 1.9% divorced. The results are explained in Table 1.

### 3.2. Symptoms of the Parkinson’s Disease

**Figure 1:**
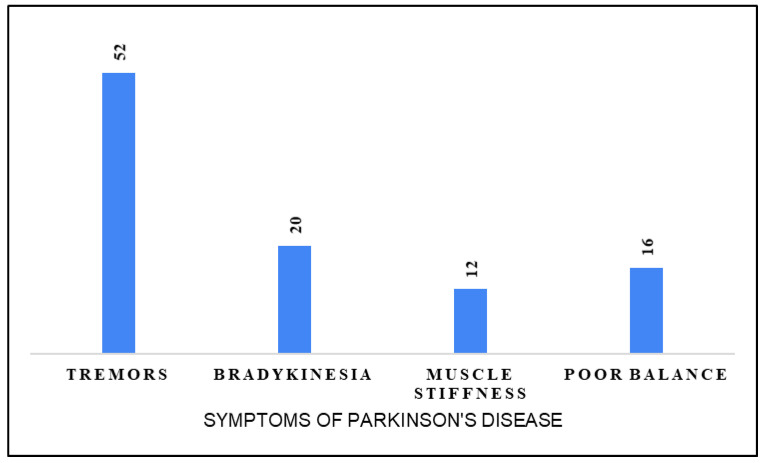
Symptoms of Parkinson’s disease.

Figure 1 illustrates the distribution of reported symptoms of Parkinson’s disease among participants. Tremors were the most recognized symptom (52%), followed by bradykinesia (20%). Poor balance was identified by 16% of respondents, while muscle stiffness was the least recognized symptom (12%). These findings highlight varying levels of awareness regarding Parkinson’s disease symptoms, emphasizing the need for targeted educational interventions.

### 3.3. Knowledge of Parkinson’s Disease

The study assessed participants’ knowledge of Parkinson’s disease, revealing that 68.3% had heard of the condition, yet 42.8% rated their knowledge as poor. While 40.9% believed Parkinson’s was curable, 47.1% were uncertain. Regarding causes, 41.6% attributed it to environmental factors, while 33.6% cited brain injuries. Over half (52.8%) were unaware of the diagnostic methods, and 59.5% correctly identified old age as the most common onset period. Social stigma was acknowledged by 35%, while 39.4% were uncertain. Most respondents (66.3%) believed Parkinson’s was difficult to treat, though 71.4% agreed that patients could lead fulfilling lives with proper treatment. These results are explained in Table 2.

### 3.4. Awareness About Parkinson’s Disease

The study examined public attitudes toward Parkinson’s disease, revealing that 54.4% felt comfortable interacting with affected individuals, and 70.5% believed they should engage in social activities. A strong majority (81.9%) supported the need for public awareness campaigns, and 67.7% recognized caregivers as essential in disease management. Nearly all respondents (95.8%) expressed interest in learning more about Parkinson’s. When suspecting someone had Parkinson’s, 84.1% would encourage medical consultation. Additionally, 79.4% were willing to participate in awareness campaigns, with the internet (52.5%) and television (24.5%) identified as key information sources. These results are discussed in Table 3.

### 3.5. Practices Regarding Parkinson’s Disease

The results demonstrate that 84.1% of participants would advocate for a suspected Parkinson’s patient to consult a physician, whereas 79.4% expressed a willingness to engage in awareness initiatives. The internet (52.5%) and television (24.5%) were recognized as the most effective resources for enhancing awareness, succeeded by healthcare professionals (12.0%) and friends and family (10.8%). The results underscore the public’s readiness to participate in Parkinson’s awareness campaigns and the significance of digital and media-oriented educational programs. The results are elucidated in Table 4.

### 3.6. Association of Knowledge by Characteristic Variables

The results show that gender, age, education, occupation, income, and region had no statistically significant impact on the measured variable. However, marital status showed a significant difference (*p* = 0.03), with divorced individuals reporting the highest mean score (20.49 ± 2.3), followed by married (19.98 ± 2.9) and single participants (19.69 ± 3.4).

Additionally, employed individuals had a slightly higher mean score (20.89 ± 2.8) compared to other occupational groups (*p* = 0.05). These findings suggest that marital status and employment may have an influence, while other demographic factors show minimal variation. These results are explained in Table 5. 

### 3.7. Association of Attitude by Characteristic Variables

The analysis indicates significant differences in scores based on gender (*p* = 0.01), age (*p* = 0.03), occupation (*p* = 0.02), and marital status (*p* = 0.01). Males (7.89 ± 1.5) scored higher than females (7.57 ± 1.5). Age-wise, participants aged 40–49 had the highest scores (8.24 ± 1.5), while those above 60 had the lowest (7.10 ± 1.1). Unemployed individuals (8.01 ± 1.5) scored higher than other occupational groups. Marital status also influenced scores, with single participants scoring the highest (8.16 ± 1.3), followed by divorced (7.95 ± 1.2) and married (7.60 ± 1.6). Other demographic factors, including education, income, and region, showed no significant differences. The results are explained in Table 6.

### 3.8. Association of Practice by Characteristic Variables

The results reveal significant differences in scores based on gender (*p* = 0.01), age (*p* = 0.01), education (*p* = 0.01), and marital status (*p* = 0.01 **). Males (4.16 ± 0.7) scored higher than females (3.93 ± 0.7). Participants aged 50–59 had the highest scores (4.53 ± 0.8), while those aged 21–29 had the lowest (3.85 ± 0.6). Education level also influenced scores, with high school graduates scoring the highest (4.51 ± 0.7). Marital status played a role, as single (4.61 ± 0.8) and divorced (4.58 ± 0.3) participants scored higher than married individuals (3.81 ± 0.6). No significant differences were observed for occupation, income, or region. These results are explained in Table 7.

## 4. Discussion

This study sought to evaluate the knowledge, attitudes, and practices (KAP) concerning Parkinson’s disease (PD) among individuals from diverse sociodemographic backgrounds. The results indicate substantial correlations between several demographic variables and the degrees of knowledge and awareness regarding PD. In the current study, male participants exhibited a superior mean knowledge score (4.16 ± 0.7) relative to female participants (3.93 ± 0.7), with a statistically significant difference (*p* = 0.01). This finding corresponds with a study conducted in Singapore, which indicated that male gender was linked to superior knowledge of PD [11,12]. However, this contrasts with research from sub-Saharan Africa, where no significant gender differences in PD knowledge were observed [13,14]. The identified gender disparity in knowledge scores may be ascribed to many variables. Males are more predisposed to developing Parkinson’s disease, potentially leading to heightened exposure to information on the condition via personal experience or social networks. Furthermore, societal roles and obligations may affect health-seeking behaviors and information gathering variably for men and women.

Age significantly influenced knowledge of Parkinson’s disease. Participants aged 50–59 years had the highest mean knowledge score (4.53 ± 0.8), whilst those aged 21–29 years demonstrated the lowest (3.85 ± 0.6), with a statistically significant difference (*p* = 0.01). This tendency aligns with data from Lebanon, where advanced age correlated with increased knowledge levels of PD [15]. One possible explanation for this disparity is that PD predominantly affects older adults, with approximately 1% of individuals over 60 years old being diagnosed with the disease [16]. Consequently, older individuals may have more exposure to PD through personal experience, social circles, or media targeted at their age group, leading to increased knowledge about the condition. Conversely, a study in rural and urban communities in the United States found that younger individuals had better knowledge of PD [17]. This difference is likely due to geographical and cultural factors, including variations in health education, media exposure, and healthcare access. In some regions, older individuals may have greater exposure to PD through personal or family experiences, whereas younger populations in more developed countries might have better access to digital health information.

Participants with high school education had the highest mean knowledge score (4.51 ± 0.7), while those with no formal education had the lowest (3.89 ± 0.5), with a significant difference (*p* = 0.01). This finding is in line with research indicating that higher education levels correlate with a better understanding of PD. This result aligns with previous research indicating that higher educational attainment is associated with increased awareness and knowledge of PD. For instance, a national population-based survey in South Korea found that subjects who had completed more than 12 years of education showed more awareness of PD [16]. Similarly, a study assessing disease-specific knowledge among individuals with PD reported that 52% of respondents had completed more than 12 years of education, suggesting a correlation between higher education levels and PD knowledge [18]. However, the ADHESON study in Europe did not find a significant association between educational level and adherence to PD treatment [19].

Regarding attitudes towards PD, male participants exhibited a higher mean attitude score (7.89 ± 1.5) compared to female participants (7.57 ± 1.5), with a significant difference (*p* = 0.01). This finding contrasts with previous research indicating that women often report greater psychological distress and a more negative self-image in the context of PD. For instance, studies have shown that women with PD experience higher levels of psychological distress and poorer health-related quality of life compared to men. Additionally, 61% of women reported that their PD negatively impacts their self-image. Age was also significantly associated with attitudes towards PD [20]. Participants aged 40–49 years had the highest mean attitude score (8.24 ± 1.5), while those aged over 60 years had the lowest (7.10 ± 1.1), with a significant difference (*p* = 0.03). This finding contrasts with research from Lebanon, which found no significant association between age and attitudes towards PD. Marital status influenced attitudes towards PD, with single participants having a higher mean attitude score (8.16 ± 1.3) compared to married participants (7.60 ± 1.6), with a significant difference (*p* = 0.01). Contrastingly, the existing literature often highlights the challenges faced by married individuals when one partner is diagnosed with PD. For instance, research indicates that greater mutuality between spouses and PD patients is associated with better mental health for both partners, reduced caregiver burden, and improved quality of life for the spouse [21]. However, the progression of PD, especially symptoms like gait disturbances, balance issues, urinary incontinence, and motor fluctuations, can impose significant stress on marital relationships, potentially leading to decreased relationship satisfaction.

In terms of practices, male participants had a higher mean practice score (4.16 ± 0.7) compared to female participants (3.93 ± 0.7), with a significant difference (*p* = 0.01). It is important to note that PD affects men more frequently than women, with a higher prevalence observed in males [22]. This increased prevalence might make men more aware of PD and more likely to engage in related health practices. Age significantly influenced practices, with participants aged 50–59 years having the highest mean practice score (4.53 ± 0.8), while those aged 21–29 years had the lowest (3.85 ± 0.6), with a significant difference (*p* = 0.01). Educational level was also a significant factor, with participants having a high school education showing the highest mean practice score (4.51 ± 0.7). In contrast, those with no formal education had the lowest (3.89 ± 0.5), with a significant difference (*p* = 0.01). This finding aligns with the existing literature, suggesting that higher educational attainment is associated with better health-related practices and outcomes [23]. For instance, a study indicated that non-demented, nonpsychotic PD patients with more years of education experienced milder depressive symptoms and better health-related quality of life. The study suggests that education may enhance cognitive performance and provide better-coping mechanisms, leading to improved health practices and outcomes [24]. Conversely, lower educational levels have been linked to poorer health literacy and practices. Research has shown that factors such as lower education are independently associated with low health literacy in PD patients, which can adversely affect disease management and outcomes [25]. Health literacy plays a crucial role in understanding neurodegenerative diseases, influencing early detection, disease management, and treatment adherence. Individuals with higher health literacy are more likely to recognize early symptoms, seek timely medical attention, and make informed decisions about their health, leading to improved outcomes [26].

Though statistically significant, the observed differences in knowledge, attitude, and practice scores between men and women may not be substantial enough to impact real-world clinical decision-making or patient outcomes. To establish clinical relevance, it would be useful to define a meaningful threshold beyond which differences in scores translate into tangible improvements in PD awareness, early detection, or patient care.

### Limitations

This study offers valuable insights into the knowledge, attitudes, and practices (KAP) related to Parkinson’s disease (PD) across diverse sociodemographic groups; however, the study’s cross-sectional design captures a specific moment in time, limiting the ability to infer causality or observe changes over time. Potential selection bias could affect the generalizability of the findings. Addressing these limitations in future research could enhance the understanding of KAP related to PD and inform the development of targeted interventions. We acknowledge the potential limitations of online survey methods; to ensure data integrity, we implemented several measures. The survey required unique email verification to prevent duplicate responses. CAPTCHA verification was used to distinguish human respondents from bots. The survey was distributed through professional networks and verified institutional channels to ensure responses came from the intended participants.

## 5. Conclusions

Our study indicates that the general population has moderate knowledge regarding PD. Most participants have a positive attitude regarding helping and supporting people who are suffering from PD. Educational attainment emerged as a critical determinant, with those having a high school education showing the highest mean scores in both knowledge and practices, underscoring the role of education in health literacy and proactive health behaviors. Furthermore, marital status influenced attitudes toward PD, with single participants displaying more positive attitudes than their married counterparts.

These insights highlight the necessity for targeted educational interventions and awareness programs that consider sociodemographic factors to enhance PD-related knowledge and practices. Tailoring strategies to specific groups can facilitate better disease management and improve the quality of life for individuals affected by Parkinson’s disease.

## Figures and Tables

**Table 1:** Sociodemographic characteristics of participants (*n* = 640).

Variables	Frequency (*n*)	Percentage (%)
Gender		
Male	430	67.2
Female	210	32.8
Age (years)		
<20	83	13
21–29	296	46.3
30–39	74	11.6
40–49	104	16.3
50–59	73	11.4
>60	10	1.6
Education		
No formal education	12	1.9
High School	31	4.8
Bachelors	208	32.5
Post-graduation	389	60.8
Occupation		
Student	275	43.0
Employed	74	11.6
Unemployed	254	39.7
Retired	37	5.8
Income (Saudi Riyal)		
>5000	359	56.1
5000–10,000	129	20.2
10,001–15,000	64	10.0
<15,000	88	13.8
Region		
Hafr al-Batin	462	72.2
Dammam	82	12.8
Al Khobar	41	6.4
Al Ahsa	26	4.1
Jubail	29	4.5
Marital Status		
Single	205	32.0
Married	423	66.1
Divorced	12	1.9

**Table 2:** Knowledge regarding Parkinson’s disease among participants.

Characteristics	Response
Have you heard of Parkinson’s disease before?	
Yes	437 (68.3)
No	203 (31.7)
2. How would you rate your knowledge of Parkinson’s disease?	
Very good	41 (6.4)
Good	133 (20.8)
Moderate	102 (30.0)
Poor	274 (42.8)
3. Do you think Parkinson’s disease is curable?	
Yes	262 (40.9)
No	77 (12.0)
I don’t know	301 (47.1)
4. What do you believe causes Parkinson’s disease?	
Environmental factors	266 (41.6)
Genetic factors	106 (16.6)
Brain injuries	215 (33.6)
I don’t know	53 (8.3)
5. How is Parkinson’s disease diagnosed?	
Blood test	42 (6.6)
CT scan or MRI	196 (30.6)
Clinical symptoms	64 (10.0)
I don’t know	338 (52.8)
6. At what age is Parkinson’s disease most commonly diagnosed?	
Childhood	3 (0.5)
Adolescence	14 (2.2)
Old Age	381 (59.5)
I don’t know	242 (37.8)
7. Do you think Parkinson’s disease patients face social stigma?	
Yes	224 (35.0)
No	164 (25.6)
I don’t know	252 (39.4)
8. Do you believe that individuals with Parkinson’s disease can lead fulfilling lives with proper treatment?	
Easier to treat	84 (20.0)
Harder to treat	279 (66.3)
I don’t know	58 (13.8)
9. Do you believe that individuals with Parkinson’s disease can lead fulfilling lives with proper treatment?	
Strongly Agree	178 (27.8)
Agree	279 (43.6)
Neutral	156 (24.4)
Disagree	12 9 (1.9)
Strongly disagree	15 (2.3)

**Table 3:** Awareness regarding Parkinson’s disease among participants.

Characteristics	Response
I feel comfortable interacting with someone who has Parkinson’s disease.	
Strongly Agree	12 (1.9)
Agree	348 (54.4)
Neutral	276 (43.1)
Disagree	0 (0)
Strongly disagree	4(0.6)
2. I think that people with Parkinson’s disease should be encouraged to participate in social activities.	
Strongly Agree	155 (24.2)
Agree	296 (46.3)
Neutral	188 (29.4)
Disagree	0 (0)
Strongly disagree	1 (0.2)
3. I feel that more public awareness campaigns are needed to educate people about Parkinson’s disease.	
Strongly Agree	305 (47.7)
Agree	219 (34.2)
Neutral	116 (18.1)
Disagree	0 (0)
Strongly disagree	0 (0)
4. What role do you think caregivers play in managing Parkinson’s disease?	
Very Important	433 (67.7)
Important	172 (26.9)
Neutral	35 (5.5)
5. I would be interested in learning more about Parkinson’s disease and how to support those affected by it.	
Yes	613 (95.8)
No	9 (1.4)
I don’t know	18 (2.8)

**Table 4:** Practices regarding Parkinson’s disease among participants.

Characteristics	Response
If you suspected someone had Parkinson’s disease, what would you do?	
Discuss it with their family	58 (9.1)
Encourage them to visit a doctor	538 (84.1)
Do nothing	44 (6.9)
2. Would you participate in a Parkinson’s disease awareness campaign?	
Yes	508 (79.4)
No	132 (20.6)
3. What resources do you think would be most helpful in increasing awareness about Parkinson’s disease?	
Healthcare Professionals	77 (12.0)
Internet	336 (52.5)
Books	1 (0.2)
Television	157 (24.5)
Friends and family	69 (10.8)

**Table 5:** Association between knowledge and sociodemographic characteristics of participants.

Variables		
Gender		
Male	19.73 ± 2.8	0.3
Female	19.95 ± 3.0	
Age (years)		
<20	19.72 ± 3.0	0.1
21–29	19.62 ± 3.1	
30–39	20.61 ± 2.7	
40–49	19.93 ± 2.4	
50–59	19.57 ± 2.8	
>60	19.98 ± 2.3	
Education		
No formal education	20.81 ± 3.5	0.4
High School	20.05 ± 2.0	
Bachelors	19.62 ± 2.8	
Post-graduation	10.85 ± 3.0	
Occupation		
Student	19.52 ± 3.1	0.05
Employed	20.89 ± 2.8	
Unemployed	19.80 ± 2.7	
Retired	19.65 ± 2.7	
Income (Saudi Riyal)		
>5000	19.88 ± 3.0	0.5
5000–10,000	19.48 ± 3.1	
10,001–15,000	19.81 ± 2.6	
<15,000	19.89 ± 2.4	
Region		
Hafr al-Batin	19.76 ± 2.9	
Dammam	19.76 ± 2.5	
Al Khobar	20.1 ± 3.3	
Al Ahsa	19.85 ± 3.8	
Jubail	19.95 ± 3.2	
Marital Status		
Single	19.69 ± 3.4	0.03
Married	19.98 ± 2.9	
Divorced	20.49 ± 2.3	

**Table 6:** Association between awareness and sociodemographic characteristics of participants.

Variables		
Gender		
Male	7.89 ± 1.5	0.01 *
Female	7.57 ± 1.5	
Age (years)		
<20	7.64 ± 1.6	0.03 **
21–29	7.61 ± 1.5	
30–39	7.86 ± 1.3	
40–49	8.24 ± 1.5	
50–59	8.02 ± 1.5	
>60	7.10 ± 1.1	
Education		
No formal education	7.90 ± 1.5	0.2
High School	8.29 ± 1.4	
Bachelors	7.72 ± 1.8	
Post-graduation	7.77 ± 1.3	
Occupation		
Student	7.63 ± 1.6	0.02 **
Employed	7.66 ± 1.3	
Unemployed	8.01 ± 1.5	
Retired	7.63 ± 1.4	
Income (Saudi Riyal)		
>5000	7.71 ± 1.5	0.3
5000–10,000	7.76 ± 1.5	
10,001–15,000	7.98 ± 1.5	
<15,000	7.99 ± 1.5	
Region		
Hafr al-Batin	7.86 ± 1.5	0.3
Dammam	7.55 ± 1.4	
Al Khobar	7.79 ± 1.8	
Al Ahsa	7.65 ± 1.3	
Jubail	7.44 ± 1.4	
Marital Status		
Single	8.16 ± 1.3	0.01 **
Married	7.60 ± 1.6	
Divorced	7.95 ± 1.2	

* Significant difference using *t*-test, ** Significant difference sunning ANOVA test.

**Table 7:** Association between practice and sociodemographic characteristics of participants.

Variables		
Gender		
Male	4.16 ± 0.7	0.01 *
Female	3.93 ± 0.7	
Age (years)		
<20	4.00 ± 0.6	0.01 **
21–29	3.85 ± 0.6	
30–39	4.28 ± 0.7	
40–49	4.37 ± 0.9	
50–59	4.53 ± 0.8	
>60	4.00 ± 02	
Education		
No formal education	3.89 ± 0.5	0.01 **
High School	4.51 ± 0.7	
Bachelors	4.21 ± 0.8	
Post-graduation	3.99 ± 0.7	
Occupation		
Student	3.85 ± 0.6	0.2
Employed	4.31 ± 0.6	
Unemployed	4.21 ± 0.9	
Retired	4.46 ± 0.8	
Income (Saudi Riyal)		
>5000	3.93 ± 0.6	0.1
5000–10,000	4.24 ± 0.7	
10,001–15,000	4.38 ± 0.9	
<15,000	4.39 ± 0.8	
Region		
Hafr al-Batin	4.18 ± 0.6	0.3
Dammam	3.76 ± 0.1	
Al Khobar	3.75 ± 0.9	
Al Ahsa	4.04 ± 0.2	
Jubail	3.98 ± 0.8	
Marital Status		
Single	4.61 ± 0.8	0.01 **
Married	3.81 ± 0.6	
Divorced	4.58 ± 0.3	

* Significant difference using *t*-test, ** Significant difference sunning ANOVA test.

## Data Availability

All data are reported in the manuscript.

## Abbreviations

The following abbreviations are used in this manuscript:

PD	Parkinson’s disease
KAP	Knowledge, attitude, and practice
